# The Multifaceted Antimicrobial Profile of Piperine in Infectious Disease Management: Current Perspectives and Potential

**DOI:** 10.3390/ph18101581

**Published:** 2025-10-19

**Authors:** Aristodemos-Theodoros Periferakis, Grigorios-Marios Adalis, Argyrios Periferakis, Lamprini Troumpata, Konstantinos Periferakis, Christiana Diana Maria Dragosloveanu, Ana Caruntu, Ilinca Savulescu-Fiedler, Serban Dragosloveanu, Andreea-Elena Scheau, Ioana Anca Badarau, Cristian Scheau, Constantin Caruntu

**Affiliations:** 1 Faculty of Medicine, The “Carol Davila” University of Medicine and Pharmacy, 050474 Bucharest, Romania; 2 Elkyda, Research & Education Centre of Charismatheia, 17675 Athens, Greece; 3 Akadimia of Ancient Greek and Traditional Chinese Medicine, 16675 Athens, Greece; 4 Pan-Hellenic Organization of Educational Programs (P.O.E.P.), 17236 Athens, Greece; 5 Department of Ophthalmology, Faculty of Dentistry, The “Carol Davila” University of Medicine and Pharmacy, 020021 Bucharest, Romania; 6 Department of Ophthalmology, Clinical Hospital for Ophthalmological Emergencies, 010464 Bucharest, Romania; 7 Department of Oral and Maxillofacial Surgery, “Carol Davila” Central Military Emergency Hospital, 010825 Bucharest, Romania; 8 Department of Oral and Maxillofacial Surgery, Faculty of Dental Medicine, Titu Maiorescu University, 031593 Bucharest, Romania; 9 Department of Internal Medicine, The “Carol Davila” University of Medicine and Pharmacy, 050474 Bucharest, Romania; 10 Department of Internal Medicine and Cardiology, Coltea Clinical Hospital, 030167 Bucharest, Romania; 11 Department of Orthopaedics and Traumatology, The “Carol Davila” University of Medicine and Pharmacy, 050474 Bucharest, Romania; 12 Department of Orthopaedics, “Foisor” Clinical Hospital of Orthopaedics, Traumatology and Osteoarticular TB, 021382 Bucharest, Romania; 13 Department of Radiology and Medical Imaging, “Foisor” Clinical Hospital of Orthopaedics, Traumatology and Osteoarticular TB, 021382 Bucharest, Romania; 14 Department of Physiology, The “Carol Davila” University of Medicine and Pharmacy, 050474 Bucharest, Romania; 15 Department of Dermatology, “Prof. N.C. Paulescu” National Institute of Diabetes, Nutrition and Metabolic Diseases, 011233 Bucharest, Romania

**Keywords:** black pepper, piperine, antibacterial, antifungal, antiparasitic, antiviral, traditional medicine

## Abstract

Piperine is an alkaloid found in plants of the genus Piper, and particularly in *P. nigrum*. This compound has been under extensive research lately for its antimicrobial, antiviral, and also anti-inflammatory, anti-oxidant, anticancer, and positive metabolic properties. Regarding its antibacterial applications, current data show that piperine is effective against *Bacillus sphaericus*, *Bacterioides fragilis*, *Escherichia coli*, *Mycobacterium tuberculosis*, *Staphylococcus aureus*, *Streptococcus mutans*, *Pseudomonas aeruginosa*, and *Vibrio cholerae*; its antifungal potency is exerted against *Candida albicans* and members of the Aspergillus family; antiviral activity has been documented against MERS-CoV, SARS-CoV2, EBOV, DENV, HCV, ZKV, and HPIV; and antiparasitic activity against Leishmania spp., Plasmodium spp., *Trichomonas vaginalis*, and Trypanosoma spp. While such applications are promising, more research is required to elucidate the mechanisms of action and to discover new ways of administration.

## 1. Introduction

The World Health Organization (WHO) reports a continued increase in antimicrobial resistance in recent years [1]. Novel antimicrobial agents are necessary to counter this emerging trend [2]; at the same time, there is also a recognized increasing difficulty in the treatment of certain viral infections [3].

While various applications for phytochemicals have been described, numerous research efforts are currently centered around the effectiveness of plant-derived substances in their antimicrobial and antiviral roles [4,5,6,7,8,9,10,11,12,13,14,15,16,17]. Taking advantage of relevant research in ethnobotany, it is hoped that by examining and verifying reported traditional uses of plants, plant-derived substances can be used to increase the effectiveness of current antimicrobial and antiviral treatments or even become, sometime in the future, frontline treatments of their own.

**Figure 1:**
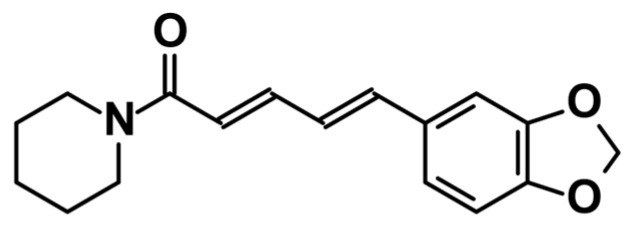
Chemical structure of piperine.

Piperine, the focus of this review, is predominantly extracted from black pepper (*Piper nigrum*) of the Piperaceae family, one of the most ancient plant families in tropical regions (Figure 1) [18]. There are other plants of this family like *Piper longum* [19] which contain piperine, but *P. nigrum* yields the highest concentration.

There are numerous ethnomedical uses of black pepper reported and a lot of research has also been performed, using modern methods, on the bioactivity and properties of its extracts [19]. Piperine, along with some volatile substances, is mostly responsible for the distinctive flavor, spiciness, and pungency of black pepper [20]. Piperine itself, perhaps the most important bioactive compound of black peppercorns, is characterized by anti-inflammatory, anticancer, anti-oxidant, analgesic, antidiabetic, and antilipidemic properties, among others [21]. The essential oil of black pepper has a number of health-related beneficial effects [22,23], while some volatile components exhibit insecticidal properties [24,25].

This review provides an integrative and comparative synthesis of piperine’s antimicrobial, antifungal, antiviral, and antiparasitic properties, bridging data from in vitro, in silico, and limited in vivo studies. The novelty of our paper is the approach of covering all pathogen classes and thoroughly analyzing effective concentrations, mechanisms of action, and translational barriers, including bioavailability and toxicity. The manuscript offers a critical, evidence-based perspective that connects traditional use of *Piper nigrum* with modern pharmacological validation, highlighting piperine’s potential as a broad-spectrum antimicrobial candidate.

## 2. Antibacterial Properties of Piperine

There is a recent scientific endeavor looking into the antibacterial potential of plant-derived compounds and metabolites [26]. A number of research efforts have been carried out focusing on piperine (Table 1).

### 2.1. Antibacterial Activity Against Bacillus *spp.*

These bacteria are rarely pathogenic, with a few exceptions [39]. Piper-longuminine, a chemical isolate from *Piper longum*, and piperine were shown to be harmful to this bacteria, exhibiting a minimum inhibitory concentration (MIC) of 25 mg/mL [27].

### 2.2. Antibacterial Activity Against Bacterioides fragilis

This bacterium is one of the many colonizers of the human gut, but a few strains are implicated in colorectal cancer [40]; on the other hand, it is implicated in potentially severe extraintestinal infections [40]. Piperine was found to have inhibitory action against *Bacteroides fragilis* at concentrations of 0.10 mg/mL [28].

### 2.3. Antibacterial Activity Against Escherichia coli

*Escherichia coli* is a physiological colonizer of the gastrointestinal tract which may become pathogenic in immunocompromised hosts [41]. The most frequent *E. coli* infections are those of the urogenital and gastrointestinal tract, while in neonates it can cause meningitis [42].

To ascertain the effectiveness of piperine against this pathogen, and its potential interaction with antibiotics, Dusane et al. [29] cultured uropathological species of *E. coli*. Piperine by itself did not show promising results in inhibiting the growth of bacteria, unless found in relatively high concentrations of 50 μg/mL. In lower concentrations however, piperine was found to decrease the expression of the genes that create the bacterial flagella; it enabled ciprofloxacin and azithromycin to more easily penetrate the created biofilm and inhibit bacterial growth [29].

### 2.4. Antibacterial Activity Against Helicobacter pylori

*Helicobacter pylori* is a common bacterium associated with gastric cancer, gastritis, and ulcers, while natural compounds may help reduce the risk of these conditions [30,31]. Another research effort, this time by Tharmalingam et al. [30], found that at an IC_50_ of 115 μΜ, piperine suppressed *H. pylori* adhesion to gastric adenocarcinoma cells and the expression of the flagellar flhA and flgE genes, thus reducing motility. It was also observed that treatment of gastric cells with piperine restrained the entry of certain Helicobacter virulence factors into cells, decreased its adhesion potential to cells, and reduced oncogenesis potential via diminished β-catenin translocation into the cell nucleus [31]. While the MIC was higher than the toxicity limit for the given cell type, sub-MICs were effective against the bacterium. Finally, based on the research of Toyoda et al. [32], piperine was found to reduce the expression of interleukin 1β, interferon γ, and interleukin 6, along with that of inducible nitrogen oxide synthase (iNOS).

### 2.5. Antibacterial Activity Against Mycobacterium tuberculosis

*M. tuberculosis* is the most well-known pathogen of this genus, being the causative agent of tuberculosis [43]. Despite the existence of a vaccine and individualized antibiotic treatment schemes, there has been an emergence of multi-resistant [44], extremely resistant, and total-resistant strains, which are predicted to be a cause of mortality for millions of people in the next decades [45].

Cell-mediated immunity, where T_h1_ lymphocytes have a key role, is mostly implicated in defense against *M. tuberculosis* infections. Based on that, the research team of Sharma et al. [33] revealed that piperine exhibited an important increase in immune response by inducing T_h1_ lymphocyte production. This effect was exerted at a 1 μg/mL dose of piperine while higher doses showed negative effects on the proliferation of lymphocytes. On the contrary, both 1 and 10 μg/mL of piperine were proven to upregulate interferon-γ and interleukin-2 in a dose-dependent manner. The potential of *Piper nigrum* as a treatment at least for some symptoms of tuberculosis has also been mentioned by Mohamad et al. [46].

### 2.6. Antibacterial Activity Against Pseudomonas aeruginosa

*P. aeruginosa* is an opportunistic human pathogen, which can cause both localized and systemic infections [47] and is a causative agent of nosocomial infections [48]. In certain cases it may persist for decades [49], and may be associated with increased morbidity and mortality [50]. The most worrying aspect concerns reports of emerging resistance to common antibiotic therapies [51].

It must be noted that an initial study by Vázquez-Martínez et al. [52] found that neither piperine on its own nor *P. nigrum* extract could have an appreciable antibacterial effect against *P. aeruginosa*. On the other hand, Das et al. [34] demonstrated the potential of piperine to inhibit Pseudomonas-associated biofilm formation, via the accumulation of reactive oxygen species (ROS), to reduce surface hydrophobicity and bacterial motility, and the potential to affect the quorum sensing network, which is associated with the coding of several virulence genes [53]. In the case of carbapenem-resistant *P. aeruginosa*, piperine was found to reduce the expression of the MexAB-OprM efflux pumps, which was associated with the reported resistance [35].

### 2.7. Antibacterial Activity Against Staphylococcus aureus

This bacterium is a frequent colonizer of the human body that has raised major health concerns throughout the years, causing a diversity of diseases ranging from skin infections to pneumonia, abscess formation, and even sepsis [54].

Das et al. [36] analyzed the effects of piperine in various concentrations on MRSA. The results showed that the formation of the biofilm was substantially decreased by 36% and 45% in 8 and 16 μg/mL piperine solutions, respectively [36]. Also, when the bacteria were exposed to the higher amount of piperine, they also showed reduced metabolic activity by 33%. Moreover, it was observed that the bacterial expression of the icaA gene was decreased [36]. The aforementioned gene was the gene mostly responsible for the formation of the biofilm that protected the microbe [55]. These conclusions validate earlier experiments which showed piperine to be relatively effective against *S. aureus*, with an MIC of 12.5 μg/mL [27].

### 2.8. Antimicrobial Activity Against Streptococcus mutans

This commensal bacterium of the oral cavity can become pathogenic in certain circumstances, and is a prominent cause of dental caries formation [56].

Dwivedi et al. showed piperine to be active against this bacterium, having an MIC of 0.33 ± 0.02 mg/mL and a BIC (biofilm inhibitory concentration) of 0.0407 ± 0.03 mg/mL [37]. The significance of these results is increased when taking into consideration that the tested isolate was SM03, which has a very potent biofilm-formation capacity [37].

### 2.9. Antimicrobial Activity Against Vibrio cholerae

Cholera is a disease reported since ancient times and the bacterium responsible is a physiological inhabitant of aquatic ecosystems [57,58]. There are numerous pathogenic biotypes which produce different virulence factors [59]; in recent years, resistance to antibiotics is a cause for concern [60,61].

The study of Manjunath et al. [38] on the antibacterial activity of piperine extracted from white pepper against *V. cholerae*, specifically the O1 El Tor variant, found that it can inhibit, or at least reduce, bacterial growth, although the precise mechanisms require elucidation.

## 3. Antifungal Properties of Piperine

Even though a limited number of fungi are considered of medical interest compared to bacteria [62], and most of the infections they cause are not life-threatening, there exist cases in which infections with fungal species such as *Aspergillus fumigatus* and *Candida albicans* will lead to serious pathological conditions. The current research evidence on the antifungal properties of piperine is presented in Table 2.

### 3.1. Antifungal Activity Against Aspergillus *spp.*

The members of this genus are not generally considered prime suspects for fungal infections in the general population, but can be dangerous under specific circumstances [67]. While piperine had no significant effect on the growth of *A. flavus*, it was effective in inhibiting aflatoxin production at concentrations ranging from 1000 for aflatoxin G_2_ to 3000 μg/mL for aflatoxins B_1_, B_2_, and G_1_ [63]. In another study, piperine was used to synthesize a number of derivatives which were effective against *A. flavus*, *A. niger*, and *A. fumigatus* [64].

### 3.2. Antifungal Activity Against Candida albicans

*Candida albicans* is one of the most notable commensal microorganisms in the human body, being mainly found lining the mucosa of the gastrointestinal and the genitourinary tracts [68]. If the proper conditions are met, mainly regarding the host’s immune status and the microbiome composition, it may become pathogenic, causing significant morbidity and mortality [68].

Piperine has been shown to damage the membrane of *Candida albicans* cells, with the ensuing oxidative stress resulting in apoptosis [66]. Moreover, it also enhances the action of the antifungal agent fluconazole [66]. Its MIC ranged from 2.5 to 15 mg/L depending on the strains and isolates [66]. Similarly, the study by Trindale et al. [64] tested a variety of piperine derivatives against the strains ATCC-60193 and LM-92 and their MICs ranged from 256 to 1024 μg/mL^−1^. The antifungal action of piperine against different candidal albicans strains is also corroborated by the findings of Phuna et al. [65].

## 4. Antiviral Properties of Piperine

There are numerous common viral pathogens, and, as such, the burden of disease of viral infections is considerable [69,70,71,72], in spite of vaccine development [73]. This fact, coupled with the frequent absence of effective antiviral treatments, imposes the need to introduce novel antiviral agents. The current evidence on the antiviral actions of piperine are presented in Table 3.

### 4.1. Antiviral Activity Against Middle East Respiratory Syndrome-Related Coronavirus (MERS-CoV)

This pathogen has a zoonotic transmission, spreading from camels to their riders [83], and is implicated in respiratory tract infections of variable severity in people in over twenty-seven countries across the Middle East, Europe, North Africa, and Asia [84]. Piperine was shown to be able to inhibit fusion peptides of MERS-CoV and POPC/SM/CHOL liposomes, as the modeling fusion peptides were not able to increase the liposomes’ diameters [75]. Thus, its anti-fusogenic activity on membrane lipids, mediated by disordering effects, was demonstrated [75]. Piperine incorporated in nanovesicles was also potent as an antiviral and anti-inflammatory agent in MERS-CoV-challenged mice [74].

### 4.2. Antiviral Activity Against Severe Acute Respiratory Syndrome Coronavirus 2 (SARS-CoV-2)

This pathogen exhibits remarkable contagiousness, reaching pandemic status within six months of the initial outbreak in China, and can cause life-threatening infections [85,86]. Piperine’s effectiveness against this pathogen has been widely demonstrated. A notable reduction in the titer of SARS-CoV2 virions in Vero cells was achieved at 1.56–100 g/mL [75]. FP-SARS-CoV-2-mediated fusion of POPC/SM/CHOL liposomes was found to be impaired due to the action of piperine [75]. Other research has also exhibited piperine’s high affinity at the binding site of SARS-CoV-2 RBD Spro and Mpro, establishing itself as a promising candidate for a therapeutic substance, potentially as part of a stable complex formation [76]. Similarly, piperine was found to be able to bind and inhibit the action of 3CLPro, the virus’ main protease; its 2,5-dimethoxy phenyl amide analog in particular was three times more potent than rutin, a previously known natural inhibitor of that protease [77]. Furthermore, the use of piperine as an adjunct in curcumin preparations can increase the latter’s bioavailability by 2000%—a finding that becomes relevant when considering that curcumin can interfere with the binding of the spike glycoprotein of SARS-CoV-2 to its designated cellular receptor [78].

### 4.3. Antiviral Activity Against Ebola Virus (EBOV)

This virus has a zoonotic transmission, with its spread being subsequently facilitated by human-to-human transmission [87]. It is one of the most lethal and virulent pathogens [87,88]. Viral proteins are critical in the infection process because they impair the signaling of interferons, having a profound negative effect on the host’s immune response [88,89]. Piperine was found to be able to bind to the VP35 Interferon Inhibitory Domain in addition to other viral proteins, being notably superior to ribavirin in this regard [79].

### 4.4. Antiviral Activity Against Dengue Virus (DENV)

This virus is spread via mosquito bites and can cause symptoms of variable severity, from simple mild fever to a life-threatening hemorrhagic fever, which can potentially lead to dengue shock syndrome [90,91]. The pathogenesis mechanisms are a complex interaction between viral antigens and the human immune response [91]. Existing drugs target specific viral proteins, one of the most prominent among them being NS5Methyltransferaseprotein [92]. When compared to the commonly used antiviral agent ribavirin, piperine was shown to have superior binding affinity for most of the targets, exhibiting significant inhibiting capacity and drug-like properties [79].

### 4.5. Antiviral Activity Against Hepatitis C Virus (HCV)

Hepatitis C is an RNA virus that infects a significant number of patients all over the world, causing a chronic disease that can eventually lead to cirrhosis or hepatocellular carcinoma in many of these cases [93,94]. Apart from liver transplantation in advanced cases, the therapeutic approach during the initial stages consists of administering pegylated interferon and drugs such as ribavirin, simeprevir, and sofosbuvir, which inhibit viral replication [93,94]. Its effectiveness however is somewhat limited when it comes to genotypes 1 and 4 [93].

Through molecular docking analysis, piperine was found to have comparable affinity for the main viral polymerase NS5B to sofosbuvir [80]. The inhibition of this protein leads to termination of viral replication [80,95]. At the same time, piperine’s caco-2 permeability values were deemed sufficient, indicating favorable intestinal absorption [80]. Most importantly, the half-maximal inhibitory concentration (IC_50_) of piperine was 52.18 ± 3.21 μM when tested in vitro against HCV [80]. Even though piperine’s effectiveness is considered moderate when compared to sofosbuvir, whose IC_50_ is 0.06 ± 1.76 μM, its development into a more potent form could establish it as an alternative treatment [80].

### 4.6. Antiviral Activity Against Zika Virus (ZKV)

Zika virus is an arbovirus causing global concern, as its emergence in Brazil and the Americas suggests that even more regions could be at risk in the future [96,97]. Many of the cases can have subclinical manifestations or resemble other viral infections, such as influenza [96], dengue, and chikungunya [97], making the diagnosis quite challenging [96,97].

Piperine is able to bind to the viral RdRp protein, impairing its ability to replicate [98], with an effectiveness superior to that of sofosbuvir and favipiravir [81]. Moreover, piperine exhibited good pharmacokinetic properties, with its bioavailability score being higher than that of the currently used antiviral drugs [81].

### 4.7. Antiviral Activity Against Human Parainfluenza Virus (HPIV)

This virus comprises several different serotypes and is commonly implicated in cases of a wide variety of respiratory tract infections, upper and lower alike, in both children and adults [99]. Serotype 3 in particular is a notable threat for lung transplant patients, potentially causing long-lasting problems [100].

An in vitro cytotoxicity analysis revealed that different extracts of piperine showed potent action against this pathogen, with the recorded MIC ranging from 200 mcg in the case of methanolic extract of *Piper longum* to 1000 mcg in the cases of methanolic extracts of Piper nigrum and chloroform extracts of Piper nigrum [82].

### 4.8. Antiviral Activity Against Indian Vesiculovirus (VSV)

This mostly non-pathogenic virus has proven useful in antiviral vaccine research [101] and is also being looked into for treatment purposes in the field of oncology [102].

Piper nigrum was shown to be active against this virus, with chloroform extract being more effective than methanolic extract, and their MICs being 200 mcg and 600 mcg, respectively [82]. In the case of *Piper longum*, the opposite is true, with the MIC of the methanolic extract being 200 mcg and that of chloroform extract being 1000 mcg [82].

## 5. Antiparasitic Properties of Piperine

While the majority of parasitic infections are mostly dangerous in areas where they are endemic [103,104], they currently show a trend of increasing resistance [105]. There is a notable corpus of research results regarding the antiparasitic potency of piperine (Table 4).

### 5.1. Antiparasitic Activity Against Leishmania *spp.*

There is a plethora of Leishmania species, each with their own particularities when it comes to geographical distribution and animal reservoir [115]. The ability of Leishmania parasites to evade the immune system [116] emphasizes that a proper therapeutic approach is of the utmost importance.

The research of Vieira-Araújo et al. [108] showed that a mixture of 50% piperine and 50% meglumine antimoniate resulted in an IC_50_ of 2.09 ± 0.25 µg/mL against *L. infantum* promastigotes. Similarly, a combination of 25% piperine and 75% glucantime resulted in an IC_50_ of 7.25 ± 4.84 µg/mL against the parasite’s amastigote form [108]. It is worth noting that these results are better than those of the pentavalent antimony-based compounds which are commonly used as therapeutic agents [108]. Based on the tests of Chouhan et al. [107], piperine seems to be active against both the promastigotes and the amastigotes of *L. donovani* too, though the hexane and ethanolic extracts of *P. nigrum* are superior in this regard.

### 5.2. Antiparasitic Activity Against Malaria

Malaria is a disease caused by five members of the Plasmodium genus, the rest being rarely pathogenic, and it is one of the most ancient diseases known to humanity [117]. The use of artemisinin or an artemisinin-based combination therapy is the treatment of choice, but it has adverse effects, and malaria parasites have begun developing resistance [118].

When tested on mice, piperine was shown to be effective to a notable extent against *P. berghei* both prophylactically and therapeutically, particularly in regard to suppressing parasitemia and the clinical manifestations, due to its ability to alter the morphology of infected erythrocytes [111]. The greatest effects on parasitemia, at a maximum of 79.21% suppression, were exerted by doses of 40 mg/kg [111]. Piperine has also exhibited antiparasitic action against *Plasmodium falciparum* at an IC_50_ of > 200μM, though some safety concerns were raised regarding its potential risk for reproduction [110], and the concentration at which it was active was quite high. Its cytotoxicity was corroborated by Wansri et al. [77] whose research on Vero cells displayed an IC_50_ of 61.24 ± 2.83 against *P. falciparum* 3D7 and an IC_50_ of 56.67 ± 0.98 against *T. brucei rhodesiense*. Similar values were also reported by Thiengsusuk et al. [109], who noted that perhaps piperine is more effective during the first 8–12 h of the parasite’s lifecycle.

### 5.3. Antiparasitic Activity Against Trichomonas vaginalis

*Trichomonas vaginalis* is the causative agent of trichomoniasis, the most commonly contracted nonviral sexually transmitted disease [119]. This obligate extracellular parasite colonizes the human genitourinary tract, and may be symptomatic [119]. Both the extracts and essential oil derived by *P. nigrum* exhibited cytolytic effects against the trophozoites of *Trichomonas vaginalis*, their MLC being up to 100 µg/mL [112]. Notably, the viability of the trophozoites was impaired even in sub-MLC and lower concentrations [112].

### 5.4. Antiparasitic Activity Against Trypanosoma *spp.*

Trypanosoma cruzi is the causative agent of Chagas disease [120], a life-threatening condition whose list of endemic areas has been increasing as a result of migration [120]. Trypanosoma brucei rhodesiense on the other hand is implicated in approximately 5% of human African trypanosomiasis cases [121]. In general, trypanosomiasis is severe parasitic disease that is difficult to treat [122].

Piperine has been shown to be active against the epimastigote form of *T. cruzi* [113,123]. The research results of Cotinguiba et al. [114] suggested that there are piperamides which can be used against the parasite with an IC_50_ as low as 10.5 μM, better than benznidazole’s IC_50_ of 42.7 μM, though the effectiveness of piperine itself in that regard was found to be lacking. An important observation was that double bonds conjugated with a carbonyl group are essential for achieving high anti-Trypanosoma cruzi activity [114]. Furthermore, piperine’s cytotoxicity against *T. brucei* rhodesiense was affirmed by Wansri et al. [77] who calculated its half-maximal effective concentration (EC_50_) as being 56.7 ± 0.98 μM. Piperine’s potency may be mild, but these results highlight the significance of the methoxy-substituted phenyl amide scaffold of some piperine analogs which were also tested in this study, and whose effectiveness as anti-trypanosomal agents was notably superior [77].

## 6. Current Knowledge, Challenges, and Future Perspectives on the Antimicrobial and Antiviral Actions of Piperine

### 6.1. Action Mechanisms of Piperine

In general, phytochemicals exert their antibacterial [124,125], antifungal [126], antiparasitic [127], and antiviral actions [128,129] via a host of different mechanisms. Piperine exhibits a number of different mechanisms, which explain its antimicrobial and antiviral actions (Table 5).

Against bacteria, piperine exhibits growth inhibition effects [29,38], can suppress the motility and cellular adhesion potential of *E. coli* in particular [30,31], and can reduce the production of pro-inflammatory and virulence factors [32]. Upregulation of lymphocytes and interleukins explains its antibacterial effect against *M. tuberculosis* [33], while it can also inhibit the formation of biofilm from *P. aeruginosa* and *S. mutans* [34,37]. In other cases, it promotes oxidative destruction of bacterial and fungal cells [34,65,66], and can inhibit the production of aflatoxins from *A. flavus* [63].

Regarding viral infections, piperine has an anti-inflammatory and anti-oxidant effect in the case of MERS-CoV [74], while it can disorganize the surface structure and inhibit the cellular fusion for both MERS-CoV and SARS-CoV2 [75]; on SARS-CoV2, it may also inhibit viral proteins [77]. Finally, it may inhibit HCV replication and promote the death of cells infected with HPIV and VSV [82].

In regard to its antiparasitic activity, it inhibits the intracellular stage of *L. donovani* [107], may cause chemosuppression of parasitemia in the case of *P. berghei* [111], disrupts the membrane and inhibits the ATPase of *T. vaginalis* [112], and reduces the biological activity of *T. cruzi* [114].

**Figure 2:**
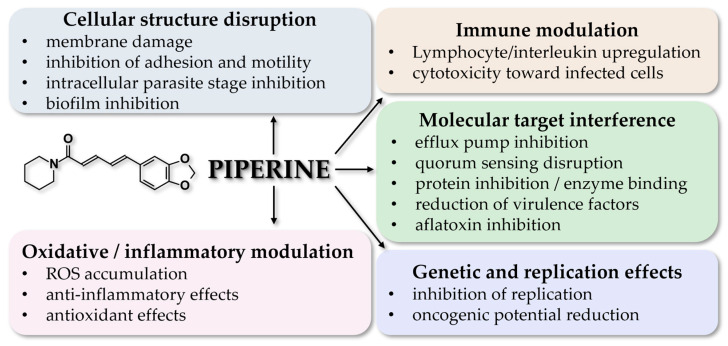
Comprehensive overview of the action mechanisms of piperine.

An overview of the action mechanisms is summarized in Figure 2.

A number of experiments also describe molecular docking mechanisms [35,76,79], and there remains a number of experiments, where even though the antimicrobial or antiviral activity of piperine has been ascertained, the precise mechanisms behind it have not been elucidated [27,28,36,64,77,106,108,110,113].

All the experiments included in our study, with the exception of the research efforts of Sharma et al. [33], Chouhan et al. [107], and Khairani et al. [111], are in vitro or in silico studies. There is, therefore, an apparent lack of in vivo data, and it is difficult to appreciate how the described in vitro effects could be translated into meaningful results in live organisms, let alone clinical settings. Issues of toxicity and methods of delivery do exist and will be discussed in the following subsection.

### 6.2. Comparative Effectiveness of Piperine

While the antibacterial, antifungal, antiparasitic, and antiviral actions of piperine are potentially potent, there remains the subject of its relative effectiveness compared to existing medication. Based on current data, it can be seen that in most cases, the pharmacological agents in use are more effective, although concentration and effectiveness vary based on the specific experimental conditions (Table 6).

Regarding the synergistic action with existing antimicrobial agents, not much data are, as of yet, available, but Mgbeahuruike et al. [141] demonstrated that piperine could be used in conjunction with rifampicin and tetracycline against *S. aureus*. It must be noted that while these results are promising, in certain cases, piperine analogs exhibit higher antibacterial activity [142] than antibiotic and therefore they might represent more promising solutions for combination with commercial antimicrobials.

### 6.3. Bioavailability of Piperine and Novel Delivery Solutions

On the subjects of delivery and bioavailability, it is indeed an issue that the high concentrations achieved in vitro would be difficult to achieve in vivo without associated toxicity and adverse effects for piperine, as well as other phytochemicals. This is an issue of concern for other phytochemicals as well [143]. At present, piperine is known to bind to plasma albumins at concentrations of about 1 μMe to 10 μM [144]. To increase its bioavailability without incurring a toxicity risk, nanoparticles can be used, with the aim of maximizing bioavailability at the needed tissues. This approach has already been tested with antibiotics [145,146,147] and recent studies indicate that nanoparticles have a theoretical antiviral potential [148,149,150]. Research into the delivery of antifungal [151,152,153] and antiparasitic agents [154,155,156] using nanoparticles is also ongoing. Specifically for phytochemicals, several barriers have to be overcome regarding water solubility, instability, and absorption in the human body [157]. Their specific pharmacokinetics create the need for specific dosing regimens [158,159]; such constraints can be addressed by employing nanoparticles and other nanoscale delivery mechanisms [160] targeting specific body tissues [161]. Specific examples of phytochemicals whose medicinal potential can be enhanced by nanotechnology are represented by allicin [162], capsaicin [163], catechins [164], curcumin [165], lycopene [166], resveratrol [167], and quercetin [168].

Aside from metal nanoparticles, lipid nanoparticles represent a viable alternative [169]. Regarding external local administration, in addition to the existing solutions of patches, creams, and gels, lipid nanoparticles of the type used in wound care are another avenue to explore [170].

Increasing the delivery of piperine to the sites of interest may also be achieved by the possibility of incorporating this compound into biomaterials, particularly 3D-printed ones used in orthopedics [171,172,173,174] and other applications [175]. Such biomaterials are being tested in terms of their antimicrobial potency in order to combat the relatively high incidence of infections related to biofilm formation [176]. Piperine could be used to supplement the effect of antibiotics and other antimicrobial substances, delivered either systemically or locally [177,178,179]. Of course, there exist a number of challenges which need to be overcome for piperine, and other antimicrobial phytochemicals, to be effectively incorporated into 3D-printed biomaterials, such as the high temperatures during some types of printing processes [180] which may diminish their antimicrobial potential [181,182], and some structural challenges. These problems can be circumvented by using different techniques depending on the necessary application [176].

It is also worth mentioning that many of the available studies on piperine do not report toxicity limits due to study type and design. This limits the ability to assess a safety margin for piperine administration, and addressing this gap is essential for future translational research.

### 6.4. Current and Future Applications

Modern biochemical and molecular research coupled with ethnobotanical practices [183,184,185,186,187,188] can be effective and complementary in phytochemical research. Piperine is one substance amongst numerous phytochemicals with proven antimicrobial and antiviral activity—the examples of capsaicin [1,143,189,190], kaempferol [191,192], quercetin [5,193,194], curcumin [195,196,197,198], coumarin [4,7,199], pinosylvin [200], and allicin [8,13,201] serve to illustrate the potency of such substances.

Black pepper presents a host of promising antibacterial activities, as presented here, and also in the relevant studies of Rosas-Piñón et al. [202] and Sharma et al. [203] concerning plants used in traditional Mexican medicine. This is important both in terms of emerging bacterial resistance and the burden of disease of bacterial infections. As seen, the antifungal activity of piperine is also promising; data also exist on the effect of *P. nigrum* extract on fungi of lesser importance, but which may prove to be of concern in the future [204]. An important number of antiparasitic and antiviral effects have been determined, particularly against significant pathogens. At the same time, other components of the essential oils of black pepper have proven active against certain pathogenic agents [205]. Piperine analogs and derivatives have also shown an antifungal potential [206,207], and the antibacterial properties of *P. nigrum* extracts oftentimes vary based on the type of extract [208].

Apart from the antimicrobial, antiparasitic, and antiviral aspects of piperine, a number of other beneficial properties of black pepper extracts have been researched thoroughly, like anti-inflammatory [209] and antidiabetic properties [210], and there is a rich record of ethnomedical black pepper uses [211,212].

## 7. Conclusions

Piperine is one of the most active compounds of black pepper (*P. nigrum*), presents a host of notable antimicrobial and antiviral activities, and is, in some cases, more potent than the agents currently in use. While the mechanisms behind such activity have been under study for quite a while, nevertheless, more research is necessary, exploring both other potential applications and ways of administration.

## Figures and Tables

**Table 1:** Antimicrobial properties of piperine, based on current research evidence, classified alphabetically by family.

Family	Genus	Species	Extracted From	Type of Experiment	Toxicity Limit	Effective Concentration	Mechanism	Year	Reference
Bacillaceae	*Bacillus*	*B. sphaericus*	*P. longum*	In vitro	n/a	25 mg/mL	n/a	2001	[27]
Bacteroidaceae	*Bacteroides*	*B. fragilis*	n/a (pure compound)	In vitro	n/a	≥0.10 mg/mL	Unclear	2020	[28]
Enterobacteriaceae	*Escherichia*	*E. coli*	n/a (pure compound)	In vitro	n/a	up to 50 μg/mL	Slight growth inhibition (high dose) to promotion of motility (lower concentration)	2014	[29]
Helicobacteraceae	*Helicobacter*	*H. Pylori*	n/a (pure compound)	In vitro	n/a	115 μΜ	Suppression of cellular adhesion and motility	2014	[30]
			n/a (pure compound)	In vitro	100 μΜ	125 μΜ	Inhibition of virulence and pro-inflammatory factors and reduction in oncogenic potential	2016	[31]
			n/a (pure compound)	In vitro	n/a	100 ppm	Suppression of pro-inflammatory factor secretion	2016	[32]
Mycobacteriaceae	*Mycobacterium*	*M. tuberculosis*	n/a (pure compound)	In vivo—mice	10 μg/mL	1 and 10 μg/mL	Upregulation of T_h1_ lymphocytes and interleukin upregulation	2014	[33]
Pseudomonadaceae	*Pseudomonas*	*P. aeruginosa*	n/a (pure compound)	In vitro	n/a	8 and 16 µg/mL	Inhibition of biofilm formation, ROS accumulation, and quorum sensing system inhibition	2023	[34]
			n/a (pure compound)	In silico	n/a	n/a	Molecular docking	2023	[35]
Staphylococcaceae	*Staphylococcus*	*S. aureus*	*P. longum*	In vitro	n/a	12.5 μg/mL	n/a	2001	[27]
		*S. aureus* (MRSA)	n/a (pure compound)	In vitro	n/a	8 and 16 μg/mL	n/a	2024	[36]
Streptococcaceae	*Streptococcus*	*S. mutans*	n/a (pure compound)	In vitro	n/a	0.33 ± 0.02 mg/mL	Inhibition of biofilm formation	2016	[37]
Vibrionaceae	*Vibrio*	*V. cholerae*	*P. nigrum*	In vitro	n/a	200 and 300 µg/mL	Growth inhibition	2022	[38]

n/a—not available.

**Table 2:** Antifungal properties of piperine, based on current research evidence, classified alphabetically by family.

Family	Genus	Species	Extracted From	Type of Experiment	Toxicity Limit	Concentration	Mechanism	Year	Reference
Aspergillaceae	*Aspergillus*	*A. flavus*	*P. nigrum*	In vitro	n/a	1000–3000 μg/mL	Aflatoxin production inhibition	2016	[63]
		*A. fumigatus*	*P. nigrum*	In vitro	n/a	n/a ^1^	Unknown	2020	[64]
		*A. niger*							
Saccharomycetaceae	*Candida*	*C. albicans*	n/a (pure compound)	In vitro	n/a	Various (alone and in mixtures)	Probably associated with oxidative stress induction	2020	[65]
			n/a (pure compound)	In vitro	n/a	5–25 mg/L	Oxidative stress induction	2021	[66]

^1^ Data only available for piperine derivatives. n/anot available.

**Table 3:** Antiviral properties of piperine, based on current research evidence, classified alphabetically by family.

Family	Genus and Species	Extracted From	Type of Experiment	Toxicity Limit	Effective Concentration	Mechanism	Year	Reference
Coronaviridae	MERS-CoV	n/a (pure compound)	In vitro	0.6 μg/mL (ΤC_50_)	14.62 ± 1.7 mcg/mL (C_max_)	Anti-inflammatory and anti-oxidant effect	2021	[74]
		n/a (pure compound)	In vitro	183.33 g/mL (IC_50_)	n/a	Surface lipid disorganization and fusion inhibition	2021	[75]
	SARS-CoV2	n/a (pure compound)	In vitro	183.33 μg/mL (IC_50_)	1.56 g/mL	Surface lipid disorganization and fusion inhibition	2021	[75]
		n/a (pure compound)	In silico	n/a	n/a	Molecular docking	2022	[76]
		*P. nigrum*	In vitro	131.67 ± 2.91 μM (EC_50_)	100 μΜ (70% inhibition)	Inhibition of 3CL^Pro^ protein	2022	[77]
		*P. nigrum*	In vitro	n/a	4.7 mg ^1^	Increase in curcumin potency	2022	[78]
Filoviridae	Ebola virus (EBOV)	*Piper nigrum*	In silico	n/a	n/a	Molecular docking	2020	[79]
Flaviviridae	Dengue Virus (DENV)	*Piper nigrum*	In silico	n/a	n/a	Molecular docking	2020	[79]
	Hepatitis C virus (HCV)	n/a (pure compound)	In vitro	n/a	52.18 ± 3.21 μM (IC_50_)	Replication inhibition—Binding to NS5B protein	2023	[80]
	Zika virus (ZKV)	*Piper nigrum*	In silico	n/a	n/a	Replication inhibition—Binding to RdRp protein	2021	[81]
Paramyxoviridae	Human parainfluenza viruses (HPIV)	*P. nigrum*, *P. longum*	In vitro—HeLa cell lines	24.18–33.43 μg/mL1 (IC_50_ at 48 h)	200–1000 ^2^ mcg	Cytotoxicity towards virus-infected cells	2017	[82]
Rhabdoviridae	Indian vesiculovirus (VSV)	*P. nigrum*, *P. longum*	In vitro—HeLa cell lines	24.18–33.43 μg/mL1 (IC_50_ at 48 h)	200–1000 ^2^ mcg	Cytotoxicity towards virus-infected cells	2017	[82]

^1^ Only concentration used in the experiment. ^2^ Different values depending on the type of extract (values refer to extracts and not to the pure compound). n/a—not available.

**Table 4:** Antiparasitic properties of piperine, based on current research evidence, classified alphabetically by species.

Family	Genus	Species	Extracted From	Type of Experiment	Toxicity Limit	Concentration	Mechanism	Year	Reference
Trypanosomatidae	*Leishmania*	*L. amazonensis*	*P. nigrum*	In vitro	n/a	15 μΜ (IC_50_)	n/a	2011	[106]
		*L. donovani*	*P. nigrum*	In vivo—BALB/c mice	n/a	14.6 μΜ (min. IC_50_ ^1^)	Inhibition of the intracellular parasite stage	2014	[107]
		*L. infantum*	*P. nigrum*	In vitro	n/a	2.09 ± 0.25 μg/mL ^2^	n/a	2018	[108]
Plasmodiidae	*Plasmodium*	*P. falciparum*	n/a (pure compound)	In vitro	n/a	59, 111.5 μM (median IC_50_ depending on strain)	Perhaps an additive/synergistic effect with other phytochemicals	2018	[109]
		*P. falciparum*	P. nigrum	In vitro	>500 μΜ (CC_50_)	>200 μΜ (IC_50_)	Unknown	2020	[110]
				In silico	n/a	n/a	Molecular docking		
		*P. berghei*	n/a (pure compound)	In vivo—Swiss Webster mice	87.0 g/mL (TC_50_) ^3^	40 mg/kg bw	Parasitemia chemosuppression	2022	[111]
		*P. falciparum*	*P. nigrum*	In vitro	131.67 ± 2.91 μM (EC_50_)	24.55 ± 1.91 μM (IC_50_)	Unknown	2022	[77]
Trichomonadidae	*Trichomonas*	*T. vaginalis*	*P. nigrum*	In vitro	No toxicity up to MLC	156, 312, 1250 μg/mL (MLCs for different extracts)	Cell membrane disruption and ATPase inhibition ^4^	2023	[112]
Trypanosomatidae	*Trypanosoma*	*T. cruzi*	*P. nigrum*	In vitro	n/a	4.91/7.36 μΜ (amastigotes/epimastigotes)	n/a	2004	[113]
		*T. cruzi*	*P. tuberculatum*	In vitro	n/a	233 μΜ (IC_50_)	Reduction in biological activity	2009	[114]
		*T. brucei rhodesiense*	*P. nigrum*	In vitro	131.67 ± 2.91 μM (EC_50_)	15.46 ± 3.09 μM (IC_50_)	Unknown	2022	[77]

^1^ Different values for different parasite stages and extracts. ^2^ Different values for different piperine combinations with other compounds (this is for piperine + meglumine antimoniate). ^3^ Toxicity value for extract. ^4^ Mechanism for *P. nigrum* extract. n/a—not available.

**Table 5:** Known antibacterial, antifungal, antiparasitic, and antiviral mechanisms of action of piperine.

Mechanism of Action	Pathogens	References
Antibacterial and Antifungal Mechanisms of Action		
Growth inhibition	*E. coli*, *P. aeruginosa*, *V. cholerae*	[29,38]
Motility alteration	*E. coli*	[30]
Cellular adhesion suppression	*H. pylori*	[30]
Reduction in oncogenic potential	*H. pylori*	[31]
Reduction in pro-inflammatory and virulence factors	*H. pylori*	[32]
Lymphocyte upregulation	*M. tuberculosis*	[33]
Interleukin upregulation	*M. tuberculosis*	[33]
Biofilm formation inhibition	*P. aeruginosa*, *S. mutans*	[34,37]
Quorum sensing system inhibition	*P. aeruginosa*	[34]
ROS accumulation/oxidative stress induction	*P. aeruginosa*, *C. albicans*	[34,65,66]
Aflatoxin production inhibition	*A. flavus*	[63]
Antiviral Mechanisms of Action		
Anti-inflammatory effect	MERS-CoV	[74]
Anti-oxidant effect	MERS-CoV	[74]
Surface structure disorganization	MERS-CoV, SARS-CoV2	[75]
Cellular fusion inhibition	MERS-CoV, SARS-CoV2	[75]
Viral protein inhibition	SARS-CoV2	[77]
Inhibition replication	HCV	[80]
Cytotoxicity towards infected cells	HPIV, VSV	[82]
Antiparasitic Mechanisms of Action		
Intracellular parasite stage inhibition	*L. donovani*	[107]
Parasitemia chemosuppression	*P. berghei*	[111]
Cellular membrane disruption	*T. vaginalis*	[112]
Parasite protein inhibition	*T. vaginalis*	[112]
Reduction in biological activity	*T. cruzi*	[114]

**Table 6:** Comparative effectiveness of piperine against used pharmacological agents (EC stands for effective concentration).

Pathogen	Drug	Drug EC	Piperine EC	Drug References	Piperine References
*B. fragilis*	Meropenem	10^6^ μg/L (MIC)	1 μg/L	[130]	[28]
*E. coli*	Trimethoprim	0.25–4 μg/mL (MIC)	50 μg/mL	[131]	[29]
	Sulfamethoxazole	4.75–76 μg/mL (MIC)			
*M. tuberculosis*	Isoniazid	0.03–0.06 mg/L (MIC)	10^4^ mg/L	[132]	[33]
	Rifampin	0.12–0.25 mg/L (MIC)			
*P. aeruginosa*	Tobramycin	1 μg/mL (MIC)	8 µg/mL	[133]	[34]
*S. aureus*	Vancomycin	0.25–2 mg/L (MIC)	8 mg/L	[134]	[36]
	Teicoplanin	0.125–4 mg/L (MIC)			
	Linezolid	0.25–4 mg/L (MIC)			
	Daptomycin	0.06–1 mg/L (MIC)			
*S. mutans*	Amoxicillin	1.95 × 10^−3^ mg/mL (MIC)	0.33 ± 0.02 mg/mL	[135]	[37]
	Penicillin	1.95 × 10^−3^ mg/mL (MIC)			
	Clindamycin	9.375 × 10^−3^ mg/mL (MIC)			
*V. cholerae*	Rifaximin	0.5–4 mg/L (MIC)	200 mg/L	[136]	[38]
*A. flavus*	Luliconazole	0.004–0.062 μg/mL (MIC)	1000–3000 μg/mL	[137]	[63]
	Lanoconazole	0.004–0.125 μg/mL (MIC)			
*C. albicans*	Fluconazole	0.25–2 mg/L (MIC)	5–25 mg/L	[138]	[66]
SARS-CoV2	Redemsivir	0.01 μΜ (EC_50_)	131.67 ± 2.91 μM	[139]	[77]
HCV	Ledipasvir	0.004–1.1 nM (EC_50_)	52.18 ± 3.21 × 10^3^ nM (IC_50_)	[140]	[80]

## Data Availability

No new data were created or analyzed in this study.

## Abbreviations

The following abbreviations are used in this manuscript:

DENV	Dengue Virus
EBOV	Ebola Virus
EC_50_	Half-maximal Effective Concentration
HCV	Hepatitis C Virus
HPIV	Human Parainfluenza Virus
IC_50_	Half-maximal Inhibitory Concentration
iNOS	Inducible Nitric Oxide Synthase
MERS-CoV	Middle East Respiratory Syndrome-related Coronavirus
MIC	Minimum Inhibitory Concentration
SARS-CoV-2	Severe Acute Respiratory Syndrome Coronavirus 2
VSV	Indian Vesiculovirus
WHO	World Health Organization
ZKV	Zika Virus
